# Higher Cerebrovascular Reactivity Predicts Better Memory Function in Older Adults Without Dementia

**DOI:** 10.1002/alz.092009

**Published:** 2025-01-03

**Authors:** Arunima Kapoor, Allison C Engstrom, Shubir Dutt, Aimée Gaubert, John Paul M Alitin, Isabel Sible, Anisa J Marshall, Fatemah Shenasa, Xingfeng Shao, Danny JJ Wang, Daniel A. Nation

**Affiliations:** ^1^ University of California, Irvine, Irvine, CA USA; ^2^ University of Southern California, Los Angeles, CA USA

## Abstract

**Background:**

Optimal cerebral blood flow is crucial to maintaining cognitive function. Cerebrovascular reactivity (CVR) is a dynamic measure of cerebrovascular function which represents the ability of cerebral blood vessels to regulate blood flow in response to vasoactive stimuli. Prior studies have demonstrated an association between impaired CVR and cognitive function in cerebrovascular and neurodegenerative conditions, including cerebral amyloid angiopathy and Alzheimer disease. However, few studies have examined the association between cerebrovascular reactivity and cognitive function in older adults prior to the onset of cognitive decline.

**Method:**

Independently living older adults (N = 111, mean age = 69.2 years; SD = 7.1; age range 55‐89 years; 29.7% male, mean education = 16.5 years; SD = 2.4) free of dementia or clinical stroke were recruited from the community and underwent brain MRI and neuropsychological assessment. Pseudo‐continuous arterial spin labeling MRI quantified whole brain cerebral perfusion during CVR to hypercapnia (vasodilation) induced by visually guided breathing exercises (15s breath holds) and indexed by capnography. We specifically examined verbal and visual memory, assessed using the RAVLT word list learning and memory, WMS‐R Logical Memory I and II, and WMS‐IV Visual Reproduction I and II.

**Result:**

Adjusting for age, sex and education, multiple regression analyses revealed a significant positive association between CVR to hypercapnia and RAVLT word list delayed recall (B = .084, 95% CI (.001, .167), p = .048), Logical Memory II (B = .240, 95% CI (.012, .468), p = .039) and Visual Reproduction II (B = .280, 95% CI (.097, .464), p = .003).

**Conclusion:**

In older adults without stroke or dementia, higher cerebrovascular reactivity is associated with better memory function. Cerebrovascular dysfunction is known to precede and contribute to the development of cognitive impairment. These findings suggest that cerebrovascular dysfunction influences cognitive performance even in preclinical stages. Future longitudinal may elucidate whether early cerebrovascular reactivity changes can predict long‐term development of dementia.

